# Asylum Construction

**Published:** 1897-12-04

**Authors:** 


### The Institutional Workshop.

### ASYLUM CONSTRUCTION.

It has been remarked that the progressive tendencies of our age are nowhere more plainly seen than in the construction and management of our public lunatic asylums, and Dr. Sibbald, the senior Commissioner in Lunacy for Scotland, has lately issued a pamphlet, illustrated by ground plans of four asylums, in which he traces the development of such institutions. As the subject is one of much importance to those entrusted with the building of asylums we purpose noticing the pamphlet at some length.

Dr. Sibbald remarks that even in bygone days the richer people could always obtain fair accommodation for their insane relatives, and no doubt this was so; but it is curious to notice that of late years the tables have almost been turned, and it is arguable that the majority of pauper lunatics are now better off than the majority of private patients. It is not spotless table linen nor luxurious fare that alone constitute the merits of an asylum, but rather careful medical treatment, trained nursing, and liberty, and in these latter we believe the pauper has the advantage.

In the evil years of the beginning of the present century, as Dr. Sibbald points out, the insane were treated in cells attached to prisons and hospitals, and even up to the middle of the century it was very rare to find that an asylum had been built for its special purpose. One of the ground plans given in this pamphlet is that of the Derby County Asylum, and it is given because it was considered a good plan less than fifty years ago and formed the model for several other asylums. With some modifications it would not be a bad plan yet for an asylum of 500 beds. That is, the general idea is good, but the details are in many points inferior.

As a more modern example a plan of the Barony Asylum at Lenzie in Scotland is reproduced. Here we notice that some of the objectionable features of the older asylums are got rid of. For instance, the communicating corridors under the level of the window sills are moved backwards clear of the front blocks, and generally, it may be said, there is more light and air throughout the buildings. On the other hand, we notice that eight of the day-rooms have only two of their sides free to the light, so that cross ventilation cannot be very well carried out; two more of the day-rooms are wedged in between the blocks and the dining-hall corridors, and two others face the north, while two only are perfectly satisfactory. There is a common dining-hall, so that patients must be collected three times a day from all parts of the asylum to their meals. We look upon this as a radically bad arrangement, and the old Derby idea of "compartments for meals" was infinitely better in principle, although it might have been that the principle was badly carried out. The Barony Asylum was, however, honourably distinguished from many—indeed, from most—asylums which preceded it. The management was excellent; the furniture was more homely, and it was to a very large extent an open-door asylum. There were no airing court walls to obstruct the views or cramp the patients in their exercise, or to recall the idea of a prison court; and we think the Barony Asylum is to be commended rather for these points than for its excellence as a plan.

Dr. Sibbald has evidently no sympathy with the mammoth asylums of England, for, while he cannot refrain from mentioning the Claybury Asylum as one of the most remarkable of its kind from certain points of view, he adds that it is "unwieldy," and here we are in full accord. Over and over again it has been pointed out in these columns that no asylum should exceed 1,000 beds, and there can be no excuse for exceeding that number, because the larger ones of the Claybury type are neither relatively cheaper to build nor more economical to manage than smaller ones. It is supposed that Claybury cost over £200 a bed to build, and it is certain that a very good asylum for 600 or 800 patients could be built for £130 a bed. The weekly rate of maintenance for the five London asylums is about 10s. 1½d. per head; that for the whole of England is 8s. 9d., so that there is a loss of something like £40,000 a year to the London ratepayers. In spite of these facts, which are quite well known, it would seem impossible for either the metropolitan authorities or the Lunacy Commissioners or the Secretary of State to review their position or alter their present policy.

A plan of the City of Glasgow Asylum at Gartloch is also given by Dr. Sibbald. It is divided into two parts—one for those patients who require constant attention, and one for those who do not. This principle has lately been a favourite in Scotland, but it is not easy to see what advantage it has over the older and more common arrangement in England, because the two parts are generally placed within a short distance of each other. The climatic and other conditions must be very similar. Moreover, a patient who to-day may not need careful supervision nor medical attention, may require both to-morrow; and we are of opinion that in any asylum not exceeding 1,000 beds the blocks or pavilions should be connected with one administrative centre. There is no good reason why the blocks should not be as fully equipped in the one case as the other, and the English system has the manifest advantage of easy supervision. The blocks at the Gartloch Asylum seem very well designed, but the asylum, taken as a whole, has two grave defects. One of these is that some of the blocks are three stories high, and the other is that there are central dining-halls instead of a dining-room for each ward, so that one of the advantages claimed for the plan by Dr. Sibbald, namely, that of each block being a home, breaks down in one of its most important points. In saying this we do not ignore the great strides made by this asylum.

(*To be continued.*)

